# Comparison of FDA Approved Kinase Targets to Clinical Trial Ones: Insights from Their System Profiles and Drug-Target Interaction Networks

**DOI:** 10.1155/2016/2509385

**Published:** 2016-07-28

**Authors:** Jingyu Xu, Panpan Wang, Hong Yang, Jin Zhou, Yinghong Li, Xiaoxu Li, Weiwei Xue, Chunyan Yu, Yubin Tian, Feng Zhu

**Affiliations:** ^1^Innovative Drug Research and Bioinformatics Group, School of Pharmaceutical Sciences and Innovative Drug Research Centre, Chongqing University, Chongqing 401331, China; ^2^School of Mathematics and Statistics, Beijing Institute of Technology, Beijing 100081, China

## Abstract

Kinase is one of the most productive classes of established targets, but the majority of approved drugs against kinase were developed only for cancer. Intensive efforts were therefore exerted for releasing its therapeutic potential by discovering new therapeutic area. Kinases in clinical trial could provide great opportunities for treating various diseases. However, no systematic comparison between system profiles of established targets and those of clinical trial ones was conducted. The reveal of probable difference or shift of trend would help to identify key factors defining druggability of established targets. In this study, a comparative analysis of system profiles of both types of targets was conducted. Consequently, the systems profiles of the majority of clinical trial kinases were identified to be very similar to those of established ones, but percentages of established targets obeying the system profiles appeared to be slightly but consistently higher than those of clinical trial targets. Moreover, a shift of trend in the system profiles from the clinical trial to the established targets was identified, and popular kinase targets were discovered. In sum, this comparative study may help to facilitate the identification of the druggability of established drug targets by their system profiles and drug-target interaction networks.

## 1. Introduction

The human kinome (defined as the protein kinase complement of the human genome) provided a starting point for full-scale understanding of protein phosphorylation in normal and disease states and for a comprehensive discovery of the kinase target [1]. Phylogenetic tree of the human kinome revealed that kinase was one of the most productive classes of established therapeutic targets [2]. According to the latest reports [3, 4], 46 drugs targeting the human kinome have received approval by the US Food and Drug Administration (FDA), which include 35 small molecular drugs, 6 monoclonal antibodies, and 5 biologics. The targets of these 46 drugs had attracted extensive attentions from many pharmaceutical companies owing to their pivotal roles in not only cancers [5–8] but also other disease indications, such as central nervous system disorder, inflammation, and ophthalmology [4]. However, the majority (37 out of 46) of approved drugs against kinase were developed for treating cancer with only a few exceptions like metformin for diabetes and tofacitinib for rheumatoid arthritis [9, 10]. Intensive efforts were thus exerted for releasing the therapeutic potential of the human kinome by discovering new therapeutic area of established targets [11] or by identifying novel target from those undiscovered kinase families [4].

As an effective new way to reveal the multifactorial nature of disease, network medicine was proposed to discover new therapeutic area for the established targets [12]. Particularly, kinase was found to be capable of regulating diverse disease indications other than cancer by pathway affiliation and network analysis of drug-kinase interactions [13]. Moreover, the accelerated identification of novel drug targets, especially the clinical trial ones, provided more opportunities for treating a variety of diseases [14, 15]. The clinical trial targets defined here refer to kinases that have not yet been utilized by FDA approved drugs but are under investigation in clinical trials. As reported, intensive efforts in the exploration of clinical trial target have dramatically extended the coverage of druggable families in the human kinome from the tyrosine kinase family to several other families like the calmodulin/calcium-regulated kinase, the glycogen synthase kinase (GSK), the cGMP-dependent protein kinase (PKG), the cAMP-dependent protein kinase (PKA), the CDC-like kinase (CLK), and the protein kinase C (PKC) [4, 10].

Although proteins in the human kinome demonstrated much closer homology relation to each other than to protein outside of kinase family, their sequence, structure, physicochemical properties, and many other characteristics vary significantly. As one of the most important properties reflecting the druggability of target, the system profile was frequently analyzed to evaluate the likelihood of a target to achieve therapeutic effects [16–18]. In particular, typical system profiles of a therapeutic target include the following: target affiliated signaling pathways, target subcellular locations, similarity proteins outside target's biochemical family, and level of sequence and structure similarities to the established drug targets [16–18]. Based on the system profiles of established drug targets, systems-level druggability rules were derived [16–18], which could be generalized as follows: targets similar to fewer human proteins outside of target family and associated with fewer human pathways tend to target drugs with reduced side-effects; efficacy drugs are more readily achieved by working on targets expressed in fewer tissues. In order to understand and evaluate the current trends in clinical trial development, it is of great interest to identify shift of trend between established targets and clinical trial ones from the system profiles' point of view. However, the system profiles of clinical trial kinase targets have not yet been analyzed, and no study of systematic comparison between the system profiles of established targets and that of clinical trial ones was conducted. Therefore, a comparison of system profiles would help to discover key factors defining the druggabilities of established targets [19–22].

In this study, a comparative analysis on the system profiles between established and clinical trial targets was conducted. Firstly, system profiles of these two types of targets were compared on 3 aspects: (1) the number of human proteins outside of the target families; (2) the number of target affiliated pathways; (3) the number of tissues the target is expressed in. Secondly, a reported combinational method predicting the promising targets by integrating multiple profiles (these system profiles, drug binding domain structural conformations, and protein physicochemical properties) of the target was further evaluated and discussed. Thirdly, the drug-target interaction networks were used to identify popular established and clinical trial kinase targets by both approved and clinical trial drugs.

## 2. Materials and Methods

### 2.1. Collection of FDA Approved and Clinical Trial Drugs Together with Their Kinase Targets

Firstly, 1,767 approved drugs were collected from the FDA official website (Drugs@FDA), and their corresponding primary therapeutic targets were matched from the Therapeutic Target Database (TTD) [3] or identified through extensive literature review (find more details in Sections 2 and 2.3), which resulted in 1,521 FDA approved drugs with 361 identifiable primary targets. Secondly, to make a comprehensive collection of clinical trial drugs, multiple resources were searched to collect more than 10,000 clinical trial drugs, which include the TTD [3], the PhRMA (http://www.phrma.org/) medicines in development, the drug pipeline reports from the websites, and annual reports of more than 150 pharmaceutical and biotechnology companies, as well as additional literature search. Thirdly, the clinical status of those clinical trial drugs was identified by the US National Institutes of Health's (NIH) ClinicalTrials.gov website (https://clinicaltrials.gov/) and the public announcements by the drug developers. As a result, ~6,000 clinical trial drugs with available clinical trial information against ~800 primary therapeutic targets were identified. Among these targets, ~500 were clinical trial targets that have not yet been utilized by approved drugs but are under investigation in clinical trials. Fourthly, the biochemical classes of established and clinical trial targets were collected from the UniProt database [23, 24]. Only drugs targeting protein kinase were analyzed in this study, which included 46 approved drugs against 25 established targets and 149 clinical trial drugs against 39 clinical trial targets.

### 2.2. System Profiles of Established and Clinical Trial Kinase Targets

Sequences of studied targets were downloaded by mapping their name to the UniProt database [23, 24]; pathway information was collected from the KEGG database [25] by crossmatching IDs of the UniProt database; tissue distribution information was collected from the TissueDistributionDBs [26] by querying using gene name of the targets. Moreover, similarity level among protein sequences were calculated by the tool of BLAST [27] which was provided by the US National Center for Biotechnology Information. Statistical comparison of system profiles were conducted by R software [28] and all figures were drawn in Microsoft Excel. In particular, the boxplot function in the basic package of R was applied in this study to draw the boxplot of system profiles among established and clinical trial (phase 3, phase 2, and phase 1) targets.

### 2.3. Identification of the Primary Therapeutic Targets of Approved and Clinical Trial Drugs

The primary therapeutic targets of approved and clinical trial drugs were identified by a well-established target validation process, which demands several key criteria [29, 30]. Firstly, targets of interest should be expressed in the disease-relevant cells or tissues. Secondly, the targets should be effectively modulated by a drug or drug-like molecule with adequate biochemical activity. Thirdly, the modulation of target in cell or animal models should ameliorate the relevant disease phenotype. Last but not least, manual literature search in PubMed [31] was used to guarantee the data quality. Only when three types of validation data were collected could the target of interest be validated as a primary one. Those three validation data types include the following: experimentally determined potency of drugs against their primary targets, observed potency of drugs against disease models linked to their corresponding targets, and the observed effects of target knockout, transgenetic, RNA interference, antibody, and antisense* in vivo* models.

## 3. Results and Discussions

### 3.1. Construction of Drug-Target Interaction Networks and Subnetworks

**Figure 1:**
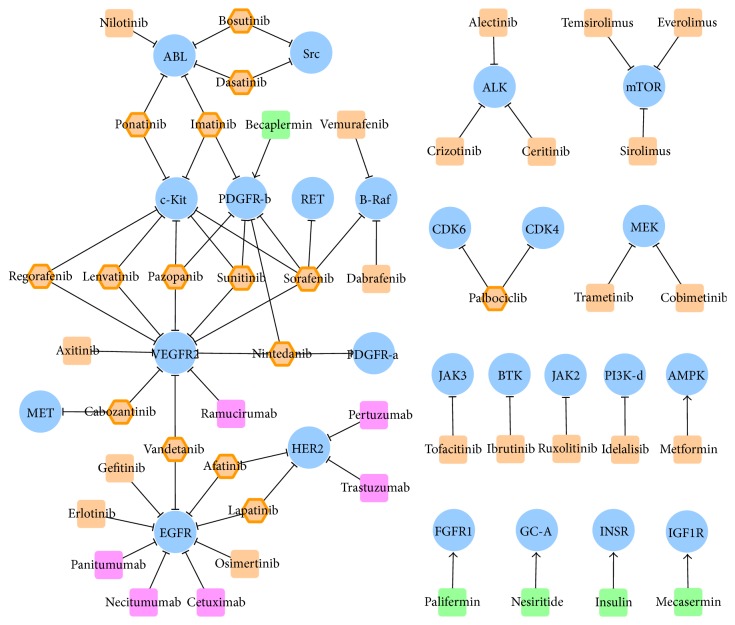
Drug-target interaction network of FDA approved drugs targeting kinase. Single target drugs were represented by round rectangle (small molecular drugs in orange, monoclonal antibodies in magenta, and biologics in green), while multitarget drugs were represented by orange hexagon and highlighted by additional orange hexagon line. All kinase targets were shown by blue ellipse. Interactions between drug and target were displayed by edges with shapes of arrow and “T” representing activation and inhibition, respectively.

**Figure 2:**
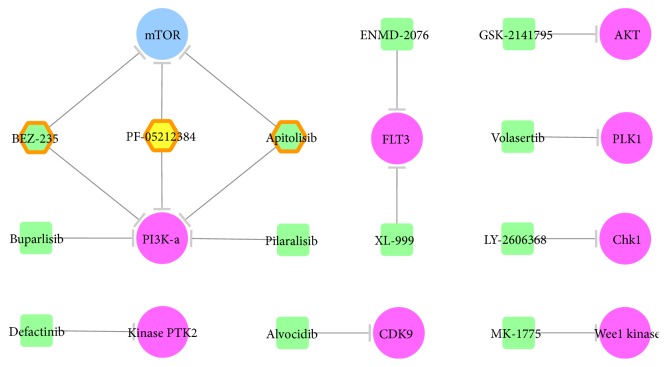
Drug-target interaction network of kinase inhibitors in clinical trial—a subnetwork of the malignant neoplasms of female genital organs (C51–C58). Single target drugs were shown as a round rectangle, while the multitarget drugs were displayed by hexagon. Colors of the drugs were defined as follows: phase 2 clinical trial drugs are in green and phase 1 clinical trial drugs are in yellow. The multitarget drugs were highlighted by an additional orange hexagon line. Established and clinical trial targets were shown by blue and violet ellipses, respectively.

Drug-target interaction networks of approved and clinical trial drugs were constructed and displayed by Cytoscape 3.3.0 [32], which is a stand-alone platform for visualizing molecular interaction networks. 46 FDA approved drugs together with their corresponding 25 targets were uploaded to and displayed in Cytoscape. As shown in Figure 1, single target drugs were shown as a round rectangle (small molecular drugs in orange, monoclonal antibodies in magenta, and biologics in green), while the multitarget drugs were displayed by orange hexagon and highlighted by additional orange hexagon line. All kinase targets were shown by blue ellipse. Interactions between drug and target were displayed by edges with shapes of arrow and “T” representing activation and inhibition, respectively. Moreover, 149 clinical trial drugs along with their 39 targets were inputted and shown in Cytoscape. The network representing drug-target interaction was provided in Figure 2 with the representation of target the same as that in Figure 1 (blue ellipse). Due to the huge number of clinical trial drugs and targets, subnetwork of specific disease class according to the International Classification of Diseases (ICD) was generated. The ICD was provided by the World Health Organization as the standard diagnostic tool for epidemiology, health management, and clinical purpose. Firstly, a specific disease class (at level 2 of ICD) named as the “malignant neoplasms of female genital organs” was selected, and clinical trial drugs and targets within this disease class were identified. Consequently, 13 drugs against 9 kinase targets were displayed [32]. As shown in Figure 2, single target drugs were shown as a round rectangle, while the multitarget drugs were displayed by hexagon. Colors of drugs were defined as phase 2 clinical trial drugs in green and phase 1 clinical trial drugs in yellow. Multitarget drugs were highlighted by additional orange hexagon lines.

### 3.2. Comparison of System Profiles between FDA Approved Kinase Targets and Clinical Trial Ones

**Figure 3:**
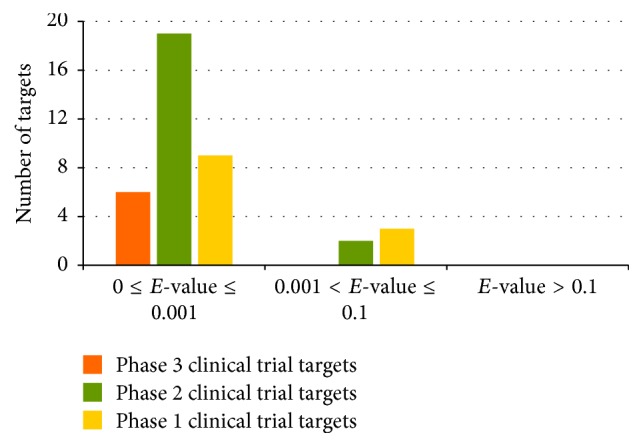
Distribution of phase 1 (yellow), phase 2 (green), and phase 3 (orange) clinical trial targets by level of similarity to established targets. The level of similarity to established targets is classified into very similar, marginally similar, and unsimilar with the BLAST *E*-values in the range of ≤0.001 and 0.001~0.1 and >0.1, respectively.

Comparison of the characteristics of the 39 clinical trial kinase targets with those of established kinase targets provides clues about common and distinguished features and shift of trends in profiles of clinical trial targets that can be retained, enhanced, or improved.  Figure 3 illustrated the distribution of phases 1, 2, and 3 clinical trial targets with respect to the level of sequence similarity to the established targets. Based on the BLAST *E*-value, the levels of similarity were classified into very similar (*E* ≤ 0.001), marginally similar (0.001 ≤ *E* ≤ 0.1), and unsimilar (*E* > 0.1). The majority of the clinical trial kinase targets (100%, 90%, and 75% of phases 3, 2, and 1) were very similar to the established ones. In addition, no clinical trial kinase target was significantly different in sequence to the established ones.

**Figure 4:**
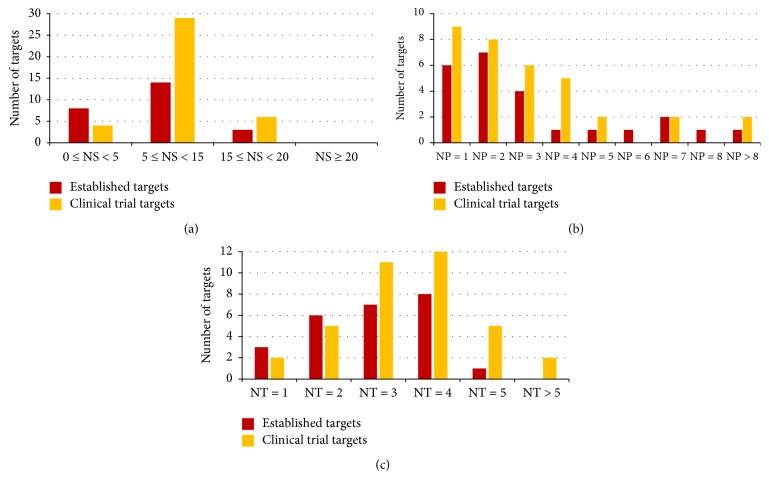
Distribution of all clinical trial kinase targets (orange) and established kinase targets (red) with respect to (a) the number of human similarity proteins (NS) outside the target family, (b) the number of human pathways (NP) the target is associated with, and (c) the number of human tissues (NT) the target is distributed in.

Figure 4 illustrated the distributions of clinical trial kinase targets and established kinase targets with respect to the number of human similarity proteins outside families of the target (Figure 4(a)), the number of target affiliated signaling pathways (Figure 4(b)), and the number of tissues that the target is distributed in (Figure 4(c)). The distribution profiles of clinical trial kinase targets were comparable to those of the established ones [17, 18]. As shown in Figure 4, 88% and 84% of the established and clinical trial targets had <15 human similarity proteins outside their target family. Furthermore, 71% and 68% of the established and clinical trial targets were affiliated to ≤3 human signaling pathways. In addition, 100% and 95% established and clinical trial targets were distributed in ≤5 human tissues. In summary, the systems profiles of vast majority of clinical trial kinase targets appear to be very similar to those of established ones [16], but the percentages of established targets obeying all three system profiles appear to be slightly but consistently higher than those of clinical trial targets.

**Figure 5:**
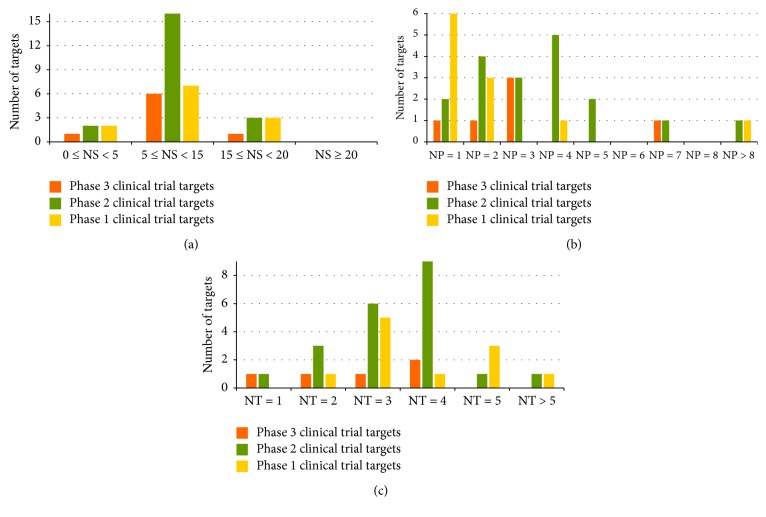
Distribution of phase 1 (yellow), phase 2 (green), and phase 3 (orange) clinical trial kinase targets with respect to (a) the number of human similarity proteins (NS) outside the target family, (b) the number of human pathways (NP) the target is associated with, and (c) the number of human tissues (NT) the target is distributed in.

Figure 5 illustrated the distributions of phase 1, phase 2, and phase 3 clinical trial kinase targets with respect to the number of human similarity proteins outside families of the target (Figure 5(a)), the number of target affiliated signaling pathways (Figure 5(b)), and the number of tissues that the target is distributed in (Figure 5(c)). As shown in figures, 86%, 85%, and 75% of phase 3, phase 2, and phase 1 clinical trial targets had <15 human similarity proteins outside their target family. Furthermore, 83%, 50%, and 82% of phase 3, phase 2, and phase 1 clinical trial targets were associated with ≤3 human pathways. In addition, 100%, 95%, and 91% of phase 3, phase 2, and phase 1 clinical trial targets were distributed in ≤5 human tissues. Consequently, percentages of phases 3, 2, and 1 clinical trial kinase targets obeying two system profiles (number of similarity proteins and tissues) appear to follow a clear descending trend, which indicates more similar profiles between established and phase 3 targets comparing to phases 2 and 1 targets.

In the meantime, the distributions of those three types of system profiles of phase 1, phase 2, and phase 3 clinical trial kinase targets and that of established targets were compared by* boxplot* (Supplementary Figure S1 in Supplementary Material available online at http://dx.doi.org/10.1155/2016/2509385). Although no significant statistical difference was observed between different clinical statuses of three types of system profiles, a shift of trend in 3 system profiles could be identified. In particular, from the established to phases 3, 2, and 1 clinical trial targets, there was a clear ascending trend of the mediums of the number of human similarity proteins outside their target family and the number of tissues that the target is distributed in. Similar ascending trend could also be observed for the number of target affiliated signaling pathways, but the medium of phase 1 targets was lower than that of phase 2 targets. In summary, as shown in Figures 4 and 5 and Supplementary Figure S1, systems profiles of vast majority of clinical trial kinase targets (especially phase 3 targets) appear to be very similar to those of established ones, which indicates that, despite extensive exploration on the innovative therapeutic target, kinases capable of entering clinical trial are those very similar to the established ones in system profiles. However, as shown in Supplementary Figure S1, there is a clear shift of trend in the system profiles from the clinical trial (phase 1 to phase 2 to phase 3) to established targets.

### 3.3. Evaluating the Performance of the Combinational Method Used for Identifying Promising Target

Majority of clinical trial targets were reported to be similar to established ones in their systems profiles [17, 18, 33–36]; target druggability may be further revealed by two more profiles: drug binding domain structural conformations [37] and protein physicochemical properties [38]. As reported, a combinational method was able to identify 50%, 25%, and 10% of the analyzed phases 3, 2, and 1 targets and 4% of nonclinical trial targets as similar to the established targets in at least 3 of the 4 profiles by systematically analyzing sequence, structural, physicochemical, and system profiles of these targets [16]. It has been 7 years since the publication of that combinational method, and it would be of great interest to evaluate its predictive performance by investigating the current developmental status of those clinical trial targets. As shown in Table 1, of the 16 phase 3 targets similar to the established ones in no less than 3 profiles [16], 5 (31%) have been approved and 6 (37%) have shown positive phase 3 results. Moreover, no positive result has been reported for 13 of the 15 phase 3 targets similar to the established ones in less than 3 profiles (with only one exception (FPTase), whose drug was filed for approval but was deemed not approvable by FDA) [16]. In particular, the corresponding phase 3 drugs of 3 targets (HSP90, squalene synthase, and FLAP) were all discontinued, and those of 5 targets (AKT, MMP-2, MMP-9, MMP-12, and sphingosine kinase) were reported with negative phase 3 results. Because of its strong predictive power reflected by the real world test in this study, the combinational method appeared to be capable of capturing target druggability by the genetic, structural, physicochemical, and system profiles [16]. Moreover, these have in turn led to the exploration of individual [17, 18, 22, 35–40] and combination of profiles [16], perspectives [41–43], and algorithms [44, 45] for* in silico* target analysis and prediction.

### 3.4. Drug-Target Interaction Networks of FDA Approved and Clinical Trial Drugs Targeting Kinase

To understand drug-target interaction of FDA approved drugs targeting kinase, network of those drugs as well as their corresponding targets was shown in Figure 1. As a widely used statistical concept in network analysis, degree was applied to assess interactions of targets and drugs. Degree of a specific node (drug or target) refers to the number of edges (interaction from other nodes) connected to this node. As shown in Figure 1, the maximum and minimum numbers of degree of approved kinase inhibitors equal 5 and 1, respectively. Particularly, 1, 4, 10, and 31 kinase inhibitors target on 5 kinases, 3 kinases, 2 kinases, and 1 kinase as their primary therapeutic targets. In particular, drug of the highest degree was sorafenib.

The maximum and minimum numbers of degree of targets equal 10 and 1, respectively. Particularly, 1, 1, 1, 1, 1, 1, 3, 2, and 14 targets were targeted by 10, 9, 7, 6, 5, 4, 3, and 2 multitarget drugs and 1 multitarget drug, respectively. The targets of substantially high degree (>8 drugs) were VEGFR2 and EGFR. As reported, VEGF and its receptors were essential in the development of the renal cell carcinoma (RCC) [46], and the inhibition of VEGFR2 could provide substantial influence on RCC's pathogenesis. In the meantime, EGFR was reported to play critical roles in and acted as primary target for non-small cell lung cancer [47], breast cancer [48], and colorectal cancer [49, 50]. Based on the network analysis, VEGFR2 and EGFR were identified as the most popular primary therapeutic kinase targets of all FDA approved drugs. Supplementary Figures S2 and S3 illustrated a comprehensive drug-target interaction network including all 46 FDA approved drugs (together with their corresponding 25 established targets) and 239 drugs in clinical trial (including 81 drugs targeting only on 17 established targets, 140 drugs targeting only on 36 clinical trial targets, and 29 multitarget drugs targeting on 13 established and 13 clinical trial targets). As shown, established targets of substantially high degree of clinical trial drugs (>10 drugs) were EGFR (26 drugs), mTOR (19 drugs), VEGFR2 (14 drugs), and IGF1R (13 drugs). In summary, EGFR and VEGFR2 were identified as the most popular established targets utilized by the highest number of both approved and clinical trial drugs, while mTOR and IGF1R were also the popular established targets with high number of drugs tested in the clinical trial.

Moreover, clinical trial targets of substantially high degree of clinical trial drugs (>10 drugs) were CDK1/2 (13 drugs), Glucokinase (13 drugs), AKT (13 drugs), and Aurora B (12 drugs). Figure 2 illustrated a subnetwork of drug-target interaction of clinical trial drugs used for treating the malignant neoplasms of female genital organs (C51–C58). Typical diseases within this class included the ovarian cancer and cervical cancer. In this disease class, the maximum degree of drugs equals 2, while the minimum is 1. In particular, 3 and 10 drugs worked on 2 primary targets and 1 primary target, respectively. BEZ-235, PF-05212384, and apitolisib were dual PI3K-alpha/mTOR inhibitors currently in phase 2 or 1 clinical trials. Take BEZ-235 as an example; its dual inhibition disturbed the PI3K/AKT/mTOR signaling pathway, leading to cell apoptosis of endometrial cancer overexpressing PI3K and mTOR [51].

The maximum number of degrees of targets equals 5, while the minimum number is 1. In particular, 1, 1, 1, and 6 targets were targeted by 5, 3, and 2 clinical trial kinase inhibitors and 1 clinical trial kinase inhibitor for treating cancers of the female genital organ, respectively. Target of the highest degree was the PI3K-alpha. The development of endometrial cancer was reported to be closely associated with the disruptions in both Wnt/beta-catenin and Akt/PI3K/mTOR pathways. Particularly, the genetic mutations in the catalytic subunit of PI3K were considered typical for endometrial cancer and were present in 26%~36% of cases [52]. Moreover, target of the second largest degree was mTOR. PI3K/mTOR pathway was frequently activated in the endometrial cancer through various genetic alterations [53], which double confirmed the pivotal roles of both targets in endometrial cancer [51]. Thus, based on network analysis, mTOR and PI3K-alpha were discovered as the most popular targets of kinase inhibitors in clinical trial for cancers of female genital organs.

## 4. Conclusion

In this study, a comparative analysis on system profiles of both targets was conducted. Moreover, a previously reported combinational method used for predicting the promising targets was discussed and evaluated. Drug-target interaction networks were used to identify popular established and clinical trial kinase targets. As a result, systems profiles of the majority of clinical trial kinase targets were identified to be very similar to those of established ones, but a shift of trend in the system profiles from the clinical trial to the established targets was identified.

## Supplementary Material

Supplementary material shows the list of drugs targeting on at least one established kinase target together with their detailed therapeutic information (Suppl. Table S1), the list of drugs targeting on at least one clinical trial kinase target together with their detailed therapeutic information (Suppl. Table S2), the distribution of 3 types of system profiles of phase 1, 2 and 3 clinical trial kinase targets and that of established targets (Suppl. Fig. S1) and a comprehensive drug-target interaction network of all 46 FDA approved drugs and 239 drugs in clinical trial (Suppl. Fig. S2, S3).

## Figures and Tables

**Table 1:** Latest development status of the previously analyzed phase 3 targets similar to established targets in sequence (A), drug binding domain structural fold (B), physicochemical features (C), and systems (D) profiles.

Target (drug previously reported to be in phase 3 trial)	Similar to established targets in combination of A, B, C, and D profiles	Targeted disease conditions	Latest development status (year of report)
CCK-A receptor (dexloxiglumide)	Combination of A, B, C, and D	Irritable bowel syndrome	Positive results in phase III trial (2007) and a large European phase III trial (2010), in talks with FDA for approval (2010)

Coagulation factor IIa (SR-123781A)	Combination of A, B, C, and D	Venous thromboembolism	Positive results in a large European phase III trial (2008)

NTRK1 (lestaurtinib)	Combination of A, B, C, and D	Acute myeloid leukemia	Lestaurtinib approved by FDA as orphan drug (2006)

5HT 3 receptor (cilansetron)	Combination of A, C, and D	Irritable bowel syndrome	Positive phase III trial results (2004), filed but withdrawn for FDA approval (2005), still in talks with MHRA and EU (2010)

Heparanase (PI-88)	Combination of A, C, and D	Hepatocellular cancer	PI-88 fast tracked by FDA (2008)

MDR 3 (LY335979)	Combination of A, C, and D	Acute myeloid leukemia	

Orexin receptor (almorexant)	Combination of A, C, and D	Sleep disorders	Positive phase III trial result (2010)

Somatostatin receptor 1 (pasireotide)	Combination of A, C, and D	Cushing's disease, renal cell carcinoma	

NK-2 receptor (saredutant)	Combination of A, C, and D	Depression	Positive phase III trial result (2007), trial discontinued (2009)

BK-2 receptor (icatibant)	Combination of A, B, and C	Hereditary angioedema, traumatic brain injuries	Positive phase III trial results (2006), icatibant approved in EU (2008)

Thrombin receptor (SCH-530348)	Combination of A, B, and C	Cardiovascular disorders	

CXCR4 (plerixafor)	Combination of A, B, and D	Non-Hodgkin's lymphoma, late-stage solid tumors	Plerixafor approved by FDA (2008)

C1 esterase (Cinryze)	Combination of A, B, and D	Hereditary angioedema	Cinryze approved by FDA (2008)

Sphingosine 1-phosphate receptor 1 (Gilenia)	Combination of A, B, and D	Multiple sclerosis	Positive phase III trial results (2008). FDA granted priority review (2010)

NPYR5 (CGP71683A)	Combination of A, B, and D	Obesity	

Plasma kallikrein (ecallantide)	Combination of A, B, and D	Hereditary angioedema	Positive phase III trial results (2007), ecallantide approved by FDA (2009)
