# Supplementary material for: Comparison of FDA Approved Kinase Targets to Clinical Trial Ones: Insights from Their System Profiles and Drug-Target Interaction Networks
Source: Biomed Res Int. 2016 Jul 28;2016:2509385. doi: 10.1155/2016/2509385 (PMC4980536; doi:10.1155/2016/2509385)

Supplementary Material for:

Comparison of FDA approved kinase targets to clinical trial ones: insights from their system profiles and drug-target interaction networks

Jingyu Xu[1,2,+], Panpan Wang[1,+], Hong Yang[1,+], Jin Zhou[1], Yinghong Li[1], Xiaoxu Li[1], Weiwei Xue[1], Chunyan Yu[1], Yubin Tian[2], Feng Zhu[1,*]

[1] Innovative Drug Research and Bioinformatics Group, School of Pharmaceutical Sciences and Innovative Drug Research Centre, Chongqing University, Chongqing 401331, China

[2] School of Mathematics and Statistics, Beijing Institute of Technology, Beijing100081, China

[+] These authors contributed equally

* Corresponding author: Feng Zhu (zhufeng@cqu.edu.cn)

Supplementary Table S1: The list of drugs targeting on at least one established kinase target together with their highest clinical trial status, code of ICD class, disease indication and their corresponding targets.

Drug Name	Highest clinical trial status	Code of ICD class	Disease indication	Target Name
Bosutinib	Approved	C00-D48	Chronic myelogenous leukemia	ABL1
Dasatinib	Approved	C00-D48	Acute lymphoblastic leukemia	ABL1
Imatinib	Approved	C00-D48	Ph+ chronic myelogenous leukemia	ABL1
Nilotinib	Approved	C00-D48	Ph+ Chronic myelogenous leukemia	ABL1
Ponatinib	Approved	C00-D48	Ph+ Chronic myelogenous leukemia	ABL1
ABL-001	Phase 1	C00-D48	Leukemia	ABL1
Rebastinib	Phase 1	C00-D48	Chronic myelogenous leukemia	ABL1
Bafetinib	Phase 2	C00-D48	Prostate cancer	ABL1
Danusertib	Phase 2	C00-D48	Chronic myelogenous leukemia	ABL1
Flumatinib	Phase 3	C00-D48	Chronic myelogenous leukemia	ABL1
Radotinib	Phase 3	C00-D48	Chronic myelogenous leukemia	ABL1
Alectinib	Approved	C00-D48	Non-small-cell lung carcinoma	ALK
Ceritinib	Approved	C00-D48	Non-small-cell lung carcinoma	ALK
Crizotinib	Approved	C00-D48	Non-small-cell lung carcinoma	ALK
Brigatinib	Phase 2	C00-D48	Non-small-cell lung carcinoma	ALK
Entrectinib	Phase 2	C00-D48	Solid tumours	ALK
Dabrafenib	Approved	C00-D48	Malignant melanoma	BRAF
Sorafenib	Approved	C00-D48	Hepatocellular carcinoma	BRAF
Vemurafenib	Approved	C00-D48	Malignant melanoma	BRAF
RAF-265	Phase 2	C00-D48	Malignant melanoma	BRAF
Ibrutinib	Approved	C00-D48	Chronic lymphocytic leukemia	BTK
Palbociclib	Approved	C00-D48	Breast cancer	CDK4/6

Afatinib	Approved	C00-D48	Non-small-cell lung carcinoma	EGFR
Cetuximab	Approved	C00-D48	Colorectal cancer	EGFR
Erlotinib	Approved	C00-D48	Non-small-cell lung carcinoma	EGFR
Gefitinib	Approved	C00-D48	Non-small-cell lung carcinoma	EGFR
Lapatinib	Approved	C00-D48	Breast cancer	EGFR
Necitumumab	Approved	C00-D48	Non-small-cell lung carcinoma	EGFR
Osimertinib	Approved	C00-D48	Non-small-cell lung carcinoma	EGFR
Panitumumab	Approved	C00-D48	Colorectal cancer	EGFR
Vandetanib	Approved	C00-D48	Thyroid cancer	EGFR
ABT-806	Phase 1	C00-D48	Solid tumours	EGFR
AMG-595	Phase 1	C00-D48	Anaplastic astrocytoma	EGFR
BIBX-1382	Phase 1	C00-D48	Cancer	EGFR
BMS-599626	Phase 1	C00-D48	Solid tumours	EGFR
CUDC-101	Phase 1	C00-D48	Breast cancer	EGFR
Imgatuzumab	Phase 1	C00-D48	Head and neck cancer	EGFR
IMGN-289	Phase 1	C00-D48	Solid tumours	EGFR
JNJ-26483327	Phase 1	C00-D48	Solid tumours	EGFR
MM-151	Phase 1	C00-D48	Solid tumours	EGFR
MR1-1	Phase 1	C00-D48	Malignant Brain Tumor	EGFR
Pelitinib	Phase 1	C00-D48	Solid tumours	EGFR
AEE-788	Phase 1/2	C00-D48	Glioblastoma	EGFR
EGF-816	Phase 1/2	C00-D48	Non-small-cell lung carcinoma	EGFR
ABT-414	Phase 2	C00-D48	Glioblastoma	EGFR
Canertinib	Phase 2	C00-D48	Breast cancer	EGFR
Duligotuzumab	Phase 2	C00-D48	Head and neck cancer	EGFR

Modotuximab	Phase 2	C00-D48	Colorectal cancer	EGFR
Matuzumab	Phase 2	C00-D48	Gastric cancer	EGFR
Sapitinib	Phase 2	C00-D48	Colorectal cancer	EGFR
ASP-8273	Phase 3	C00-D48	Non-small-cell lung carcinoma	EGFR
Dacomitinib	Phase 3	C00-D48	Non-small-cell lung carcinoma	EGFR
Icotinib	Phase 3	C00-D48	Non-small-cell lung carcinoma	EGFR
Rindopepimut	Phase 3	C00-D48	Glioblastoma	EGFR
Rociletinib	Phase 3	C00-D48	Non-small-cell lung carcinoma	EGFR
Tesevatinib	Phase 3	C00-D48	Non-small-cell lung carcinoma	EGFR
Zalutumumab	Phase 3	C00-D48	Head and neck cancer	EGFR
Afatinib	Approved	C00-D48	Non-small-cell lung carcinoma	ERBB2
Lapatinib	Approved	C00-D48	Breast cancer	ERBB2
Pertuzumab	Approved	C00-D48	Breast cancer	ERBB2
Trastuzumab	Approved	C00-D48	Breast cancer	ERBB2
BMS-599626	Phase 1	C00-D48	Solid tumours	ERBB2
CUDC-101	Phase 1	C00-D48	Breast cancer	ERBB2
Poziotinib	Phase 2	C00-D48	Breast cancer	ERBB2
Varlitinib	Phase 2	C00-D48	Breast cancer	ERBB2
Neratinib	Phase 3	C00-D48	Breast cancer	ERBB2
Palifermin	Approved	C00-D48	Oral mucositis	FGFR1
FP-1039	Phase 2	C00-D48	Mesothelioma	FGFR1
Lucitanib	Phase 2	C00-D48	Breast cancer	FGFR1
Sulfatinib	Phase 3	C00-D48	Neuroendocrine tumours	FGFR1
AVE-1642	Phase 1	C00-D48	Multiple myeloma	IGF1R
BIIB-022	Phase 1	C00-D48	Hepatocellular carcinoma	IGF1R

KW-2450	Phase 1	C00-D48	Solid tumours	IGF1R
MM-141	Phase 1	C00-D48	Hepatocellular carcinoma	IGF1R
XL-228	Phase 1	C00-D48	Leukemia	IGF1R
BMS-754807	Phase 2	C00-D48	Breast cancer	IGF1R
Cixutumumab	Phase 2	C00-D48	Cancer	IGF1R
Dalotuzumab	Phase 2	C00-D48	Neuroendocrine tumours	IGF1R
Masoprocol	Phase 2	C00-D48	Prostate cancer	IGF1R
Picropodophyllin	Phase 2	C00-D48	Non-small-cell lung carcinoma	IGF1R
Teprotumumab	Phase 2	C00-D48	Advanced Breast cancer	IGF1R
Figitumumab	Phase 3	C00-D48	Multiple myeloma	IGF1R
Ganitumab	Phase 3	C00-D48	Solid tumours	IGF1R
Linsitinib	Phase 3	C00-D48	Multiple myeloma	IGF1R
Insulin	Approved	C00-D48	Diabetes mellitus	INSR
Ruxolitinib	Approved	C00-D48	Myelofibrosis	JAK2
Tofacitinib	Approved	L00-L99	Rheumatoid arthritis	JAK3
Axitinib	Approved	C00-D48	Renal cancer	KDR
Cabozantinib	Approved	C00-D48	Thyroid cancer	KDR
Lenvatinib	Approved	C00-D48	Thyroid cancer	KDR
Nintedanib	Approved	C00-D48	Non-small-cell lung carcinoma	KDR
Pazopanib	Approved	C00-D48	Renal cancer	KDR
Ramucirumab	Approved	C00-D48	Colorectal cancer	KDR
Regorafenib	Approved	C00-D48	Colorectal cancer	KDR
Sorafenib	Approved	C00-D48	Hepatocellular carcinoma	KDR
Sunitinib	Approved	C00-D48	Gastrointestinal stromal tumours	KDR
Vandetanib	Approved	C00-D48	Thyroid cancer	KDR

CYC-116	Phase 1	C00-D48	Solid tumours	KDR
OSI-930	Phase 1	C00-D48	Solid tumours	KDR
TAK-593	Phase 1	C00-D48	Solid tumours	KDR
JI-101	Phase 1/2	C00-D48	Solid tumours	KDR
Orantinib	Phase 1/2	C00-D48	Hepatocellular carcinoma	KDR
ENMD-2076	Phase 2	C00-D48	Breast cancer	KDR
Lucitanib	Phase 2	C00-D48	Breast cancer	KDR
Motesanib	Phase 2	C00-D48	Breast cancer	KDR
XL-999	Phase 2	C00-D48	Cancer	KDR
Cediranib	Phase 3	C00-D48	Colorectal cancer	KDR
Famitinib	Phase 3	C00-D48	Colorectal cancer	KDR
Fruquintinib	Phase 3	C00-D48	Colorectal cancer	KDR
Sulfatinib	Phase 3	C00-D48	Neuroendocrine tumours	KDR
Tivozanib	Phase 3	C00-D48	Hepatocellular carcinoma	KDR
Imatinib	Approved	C00-D48	Ph+ chronic myelogenous leukemia	KIT
Lenvatinib	Approved	C00-D48	Thyroid cancer	KIT
Pazopanib	Approved	C00-D48	Renal cancer	KIT
Ponatinib	Approved	C00-D48	Ph+ Chronic myelogenous leukemia	KIT
Sorafenib	Approved	C00-D48	Hepatocellular carcinoma	KIT
Sunitinib	Approved	C00-D48	Gastrointestinal stromal tumours	KIT
Amuvatinib	Phase 1	C00-D48	Solid tumours	KIT
OSI-930	Phase 1	C00-D48	Solid tumours	KIT
Motesanib	Phase 2	C00-D48	Breast cancer	KIT
XL-820	Phase 2	C00-D48	Gastrointestinal stromal tumours	KIT
Famitinib	Phase 3	C00-D48	Colorectal cancer	KIT

Cobimetinib	Approved	C00-D48	Malignant melanoma	MAP2K1
Trametinib	Approved	C00-D48	Malignant melanoma	MAP2K1
RG-7304	Phase 1	C00-D48	Solid tumours	MAP2K1
Cabozantinib	Approved	C00-D48	Thyroid cancer	MET
Altiratinib	Phase 1	C00-D48	Solid tumours	MET
Everolimus	Approved	C00-D48	HER2-negative breast cancer	mTOR
Sirolimus	Approved	J00-J99	Lymphangioleiomyomatosis	mTOR
Temsirolimus	Approved	C00-D48	Advanced renal cell carcinoma	mTOR
AZD-8055	Phase 1	C00-D48	Solid tumours	mTOR
CC-115	Phase 1	C00-D48	Multiple myeloma	mTOR
DS-3078	Phase 1	C00-D48	Lymphoma	mTOR
DS-7423	Phase 1	C00-D48	Solid tumours	mTOR
GDC-0349	Phase 1	C00-D48	Non-Hodgkin's lymphoma	mTOR
OSI-027	Phase 1	C00-D48	Lymphoma	mTOR
PWT-33597	Phase 1	C00-D48	Solid tumours	mTOR
VS-5584	Phase 1	C00-D48	Lymphoma	mTOR
BGT-226	Phase 1/2	C00-D48	Solid tumours	mTOR
CC-223	Phase 1/2	C00-D48	Multiple myeloma	mTOR
Apitolisib	Phase 2	C00-D48	Breast cancer	mTOR
AZD-2014	Phase 2	C00-D48	Non-small-cell lung carcinoma	mTOR
Dactolisib	Phase 2	C00-D48	Solid tumours	mTOR
Gedatolisib	Phase 2	C00-D48	Acute myeloid leukemia	mTOR
LY-3023414	Phase 2	C00-D48	Endometrial cancer	mTOR
PF-4691502	Phase 2	C00-D48	Breast cancer	mTOR
Sapanisertib	Phase 2	C00-D48	Prostate cancer	mTOR

Voxtalisib	Phase 2	C00-D48	Solid tumours	mTOR
Ridaforolimus	Phase 3	I00-I99	Coronary artery restenosis	mTOR
Nesiritide	Approved	I00-I99	Congestive heart Failure	NPR1
Becaplermin	Approved	C00-D48	Skin cancer	PDGFRA
Imatinib	Approved	C00-D48	Ph+ chronic myelogenous leukemia	PDGFRA
Nintedanib	Approved	C00-D48	Non-small-cell lung carcinoma	PDGFRA
Amuvatinib	Phase 1	C00-D48	Solid tumours	PDGFRA
Crenolanib	Phase 2	C00-D48	Acute myeloid leukemia	PDGFRA
Tovetumab	Phase 2	C00-D48	Glioblastoma	PDGFRA
Olaratumab	Phase 3	C00-D48	Gastrointestinal stromal tumours	PDGFRA
Becaplermin	Approved	C00-D48	Skin cancer	PDGFRB
Imatinib	Approved	C00-D48	Ph+ chronic myelogenous leukemia	PDGFRB
Nintedanib	Approved	C00-D48	Non-small-cell lung carcinoma	PDGFRB
Pazopanib	Approved	C00-D48	Renal cancer	PDGFRB
Sorafenib	Approved	C00-D48	Hepatocellular carcinoma	PDGFRB
Sunitinib	Approved	C00-D48	Gastrointestinal stromal tumours	PDGFRB
TAK-593	Phase 1	C00-D48	Solid tumours	PDGFRB
JI-101	Phase 1/2	C00-D48	Solid tumours	PDGFRB
Crenolanib	Phase 2	C00-D48	Acute myeloid leukemia	PDGFRB
sNN-0031	Phase 2	G00-G99	Parkinson's disease	PDGFRB
Idelalisib	Approved	C00-D48	Chronic lymphocytic leukemia	PIK3CD
GSK-2269557	Phase 2	J00-J99	COPD	PIK3CD
Metformin	Approved	C00-D48	Type 2 diabetes mellitus	PRKAA
Acadesine	Phase 3	C00-D48	Myelodysplastic syndromes	PRKAA
Sorafenib	Approved	C00-D48	Hepatocellular carcinoma	RET

Bosutinib	Approved	C00-D48	Chronic myelogenous leukemia	SRC
Dasatinib	Approved	C00-D48	Acute lymphoblastic leukemia	SRC
XL-228	Phase 1	C00-D48	Acute lymphoblastic leukemia	SRC
Saracatinib	Phase 2/3	C00-D48	Breast cancer	SRC
Regorafenib	Approved	C00-D48	Colorectal cancer	TEK
CEP-11981	Phase 1	C00-D48	Solid tumours	TEK

Supplementary Table S2: The list of drugs targeting on at least one clinical trial kinase target together with their highest clinical trial status, code of ICD class, disease indication and their corresponding targets.

Drug name	Highest clinical trial status	Code of ICD class	Indication	Gene name of the target
Ascrinvacumab	Phase 2	C00-D48	Hepatocellular carcinoma	ACVRL1
ARQ-092	Phase 1	C00-D48	Solid tumours	AKT
BAY-1125976	Phase 1	C00-D48	Solid tumours	AKT
GSK-690693	Phase 1	C00-D48	Lymphoma	AKT
LY-2780301	Phase 1	C00-D48	Solid tumours	AKT
MSC-2363318A	Phase 1	C00-D48	Solid tumours	AKT
SR-13668	Phase 1	C00-D48	Cancer	AKT
Triciribine	Phase 1	C00-D48	Haematological malignancies	AKT
XL-418	Phase 1	C00-D48	Solid tumours	AKT
Afuresertib	Phase 2	C00-D48	Haematological malignancies	AKT
AZD-5363	Phase 2	C00-D48	Breast cancer	AKT
Ipatasertib	Phase 2	C00-D48	Breast cancer	AKT
MK-2206	Phase 2	C00-D48	Haematological malignancies	AKT
Uprosertib	Phase 2	C00-D48	Acute myeloid leukemia	AKT
AZD-6738	Phase 1	C00-D48	Gastric cancer	ATR
VX-970	Phase 2	C00-D48	Small cell lung cancer	ATR
AMG-900	Phase 1	C00-D48	Haematological malignancies	AURKA
CYC-116	Phase 1	C00-D48	Solid tumours	AURKA
KW-2449	Phase 1	C00-D48	Acute lymphoblastic leukemia	AURKA
XL-228	Phase 1	C00-D48	Acute lymphoblastic leukemia	AURKA
AT-9283	Phase 2	C00-D48	Acute myeloid leukemia	AURKA
Danusertib	Phase 2	C00-D48	Leukemia	AURKA

ENMD-2076	Phase 2	C00-D48	Breast cancer	AURKA
Tozasertib	Phase 2	C00-D48	Colorectal cancer	AURKA
AMG-900	Phase 1	C00-D48	Haematological malignancies	AURKB
BI-811283	Phase 1	C00-D48	Acute myeloid leukemia	AURKB
BI-831266	Phase 1	C00-D48	Solid tumours	AURKB
BI-847325	Phase 1	C00-D48	Solid tumours	AURKB
CYC-116	Phase 1	C00-D48	Solid tumours	AURKB
GSK-1070916A	Phase 1	C00-D48	Solid tumours	AURKB
TAK-901	Phase 1	C00-D48	Haematological malignancies	AURKB
AT-9283	Phase 2	C00-D48	Acute myeloid leukemia	AURKB
Danusertib	Phase 2	C00-D48	Chronic myelogenous leukemia	AURKB
Ilorasertib	Phase 2	C00-D48	Haematological malignancies	AURKB
Tozasertib	Phase 2	C00-D48	Colorectal cancer	AURKB
Barasertib	Phase 2/3	C00-D48	Acute myeloid leukemia	AURKB
Rimacalib	Phase 2	M00-M99	Rheumatoid arthritis	CaMKII
NMS-1116354	Phase 1	C00-D48	Solid tumours	CDC7
XL-413	Phase 1/2	C00-D48	Haematological malignancies	CDC7
AG-24322	Phase 1	C00-D48	Cancer	CDK1/2
AZD-5438	Phase 1	C00-D48	Solid tumours	CDK1/2
PHA-793887	Phase 1	C00-D48	Solid tumours	CDK1/2
RGB-286638	Phase 1	C00-D48	Solid tumours	CDK1/2
Roniciclib	Phase 1	C00-D48	Solid tumours	CDK1/2
SNS-032	Phase 1	C00-D48	B-cell lymphoma	CDK1/2
TG-02	Phase 1	C00-D48	Haematological malignancie	CDK1/2
AT-7519	Phase 2	C00-D48	Chronic lymphocytic leukemia	CDK1/2

Milciclib	Phase 2	C00-D48	Thymoma	CDK1/2
P-276	Phase 2	C00-D48	Head and neck cancer	CDK1/2
Seliciclib	Phase 2	C00-D48	Nasopharyngeal cancer	CDK1/2
UCN-01	Phase 2	C00-D48	Acute myeloid leukemia	CDK1/2
Dinaciclib	Phase 3	C00-D48	Chronic myelogenous leukemia	CDK1/2
Alvocidib	Phase 2	C00-D48	Acute myeloid leukemia	CDK9
GDC-0425	Phase 1	C00-D48	Lymphoma	CHEK1
GDC-0575	Phase 1	C00-D48	Lymphoma	CHEK1
PF-477736	Phase 1	C00-D48	Solid tumours	CHEK1
Rabusertib	Phase 1	C00-D48	Cancer	CHEK1
XL-844	Phase 1	C00-D48	Chronic lymphocytic leukemia	CHEK1
MK-8776	Phase 2	C00-D48	Acute myeloid leukemia	CHEK1
Prexasertib	Phase 2	C00-D48	Breast cancer	CHEK1
UCN-01	Phase 2	C00-D48	Acute myeloid leukemia	CHEK1
TCD-717	Phase 1	C00-D48	Solid tumours	CHKA
CX-4945	Phase 1	C00-D48	Multiple myeloma	CK2
CIGB-300	Phase 2/3	C00-D48	Condylomata acuminate	CK2
AV-203	Phase 1	C00-D48	Solid tumours	ERBB3
Elgemtumab	Phase 1	C00-D48	Breast cancer	ERBB3
GSK-2849330	Phase 1	C00-D48	Solid tumours	ERBB3
KTN-3379	Phase 1	C00-D48	Head and neck cancer	ERBB3
Lumretuzumab	Phase 1	C00-D48	Non-small-cell lung carcinoma	ERBB3
MM-141	Phase 1	C00-D48	Hepatocellular carcinoma	ERBB3
REGN-1400	Phase 1	C00-D48	Cancer	ERBB3
Patritumab	Phase 1/2	C00-D48	Non-small-cell lung carcinoma	ERBB3

Duligotuzumab	Phase 2	C00-D48	Head and neck cancer	ERBB3
Seribantumab	Phase 2	C00-D48	Breast cancer	ERBB3
AZD-5658	Phase 1	E00-E90	Type 2 diabetes mellitus	GCK
AZD-6370	Phase 1	E00-E90	Type 2 diabetes mellitus	GCK
AZD-6714	Phase 1	E00-E90	Type 2 diabetes mellitus	GCK
BMS-820132	Phase 1	E00-E90	Type 2 diabetes mellitus	GCK
DS-7309	Phase 1	E00-E90	Type 2 diabetes mellitus	GCK
TAK-329	Phase 1	E00-E90	Type 1 diabetes mellitus	GCK
ARRY-403	Phase 2	E00-E90	Type 2 diabetes mellitus	GCK
AZD-1656	Phase 2	E00-E90	Type 2 diabetes mellitus	GCK
LY-2608204	Phase 2	E00-E90	Type 2 diabetes mellitus	GCK
PF-4937319	Phase 2	E00-E90	Type 2 diabetes mellitus	GCK
PF-4991532	Phase 2	E00-E90	Type 2 diabetes mellitus	GCK
PSN-010	Phase 2	E00-E90	Type 2 diabetes mellitus	GCK
TTP-399	Phase 2	E00-E90	Type 2 diabetes mellitus	GCK
LY-2090314	Phase 2	C00-D48	Pancreatic cancer	GSK3A
LY-2090314	Phase 2	C00-D48	Pancreatic cancer	GSK3B
Tideglusib	Phase 2	G00-G99	Alzheimer's disease	GSK3B
CC-401	Phase 1	C00-D48	Acute myeloid leukemia	JNK
Tanzisertib	Phase 2	L00-L99	Discoid lupus erythematosus	JNK
XG-102	Phase 3	H00-H59	Ocular inflammation	JNK
GS-4997	Phase 2	I00-I99	Diabetic nephropathies	MAP3K5
Dilmapimod	Phase 2	J00-J99	COPD	MAPK14
Talmapimod	Phase 2	C00-D48	Multiple myeloma	MAPK14
VX-702	Phase 2	M00-M99	Rheumatoid arthritis	MAPK14

OTS-167	Phase 1	C00-D48	Solid tumours	MELK
PF-3758309	Phase 1	C00-D48	Solid tumours	PAK4
AR-12	Phase 1	C00-D48	Lymphoma	PDPK1
PFK-158	Phase 1	C00-D48	Solid tumours	PFKFB3
CH-5132799	Phase 1	C00-D48	Solid tumours	PI3K
CUDC-907	Phase 1	C00-D48	Diffuse large B-cell lymphoma	PI3K
DS-7423	Phase 1	C00-D48	Solid tumours	PI3K
GDC-0084	Phase 1	C00-D48	Glioma	PI3K
Omipalisib	Phase 1	C00-D48	Solid tumours	PI3K
PWT-33597	Phase 1	C00-D48	Solid tumours	PI3K
SF-1126	Phase 1	C00-D48	Neuroblastoma	PI3K
VS-5584	Phase 1	C00-D48	Lymphoma	PI3K
ZSTK-474	Phase 1	C00-D48	Solid tumours	PI3K
BGT-226	Phase 1/2	C00-D48	Solid tumours	PI3K
CLR-457	Phase 1/2	C00-D48	Solid tumours	PI3K
Apitolisib	Phase 2	C00-D48	Non-Hodgkin's lymphoma	PI3K
Dactolisib	Phase 2	C00-D48	Solid tumours	PI3K
Gedatolisib	Phase 2	C00-D48	Acute myeloid leukemia	PI3K
PF-4691502	Phase 2	C00-D48	Breast cancer	PI3K
Pictrelisib	Phase 2	C00-D48	Breast cancer	PI3K
Pilaralisib	Phase 2	C00-D48	Lymphoma	PI3K
Voxtalisib	Phase 2	C00-D48	Solid tumours	PI3K
Copanlisib	Phase 3	C00-D48	Non-Hodgkin's lymphoma	PI3K
Rigosertib	Phase 3	C00-D48	Chronic myelogenous leukemia	PI3K
BAY-1082439	Phase 1	C00-D48	Solid tumours	PIK3CA

CH-5132799	Phase 1	C00-D48	Solid tumours	PIK3CA
PWT-33597	Phase 1	C00-D48	Solid tumours	PIK3CA
MLN-1117	Phase 1/2	C00-D48	Non-small-cell lung carcinoma	PIK3CA
Alpelisib	Phase 2	C00-D48	Breast cancer	PIK3CA
LY-3023414	Phase 2	C00-D48	Endometrial cancer	PIK3CA
Sonolisib	Phase 2	C00-D48	Colorectal cancer	PIK3CA
Buparlisib	Phase 3	C00-D48	Breast cancer	PIK3CA
Taselisib	Phase 3	C00-D48	Breast cancer	PIK3CA
AZD-6482	Phase 1	I00-I99	Thrombosis	PIK3CB
AZD-8186	Phase 1	C00-D48	Breast cancer	PIK3CB
GSK-2636771	Phase 1	C00-D48	Solid tumours	PIK3CB
SAR-260301	Phase 1	C00-D48	Lymphoma	PIK3CB
Taselisib	Phase 3	C00-D48	Breast cancer	PIK3CB
AZD-1208	Phase 1	C00-D48	Acute myeloid leukemia	PIM1
PIM-447	Phase 1	C00-D48	Multiple myeloma	PIM1
AZD-1208	Phase 1	C00-D48	Acute myeloid leukemia	PIM2
PIM-447	Phase 1	C00-D48	Multiple myeloma	PIM2
AZD-1208	Phase 1	C00-D48	Acute myeloid leukemia	PIM3
PIM-447	Phase 1	C00-D48	Multiple myeloma	PIM3
TLN-232	Phase 2	C00-D48	Malignant melanoma	PKM
GSK-461364	Phase 1	C00-D48	Non-Hodgkin's lymphoma	PLK1
MK-1496	Phase 1	C00-D48	Solid tumours	PLK1
NMS-1286937	Phase 1	C00-D48	Solid tumours	PLK1
TAK-960	Phase 1	C00-D48	Acute myeloid leukemia	PLK1
BI-2536	Phase 2	C00-D48	Acute myeloid leukemia	PLK1

Volasertib	Phase 2	C00-D48	Acute myeloid leukemia	PLK1
BI-853520	Phase 1	C00-D48	Solid tumours	PTK2
PF-562271	Phase 1	C00-D48	Cancer	PTK2
VS-4718	Phase 1	C00-D48	Solid tumours	PTK2
Defactinib	Phase 2	C00-D48	Mesothelioma	PTK2
GSK-2256098	Phase 2	C00-D48	Meningioma	PTK2
INS-117548	Phase 1	H00-H59	Glaucoma	ROCK2
KD-025	Phase 2	J00-J99	Plaque psoriasis	ROCK2
ABC-294640	Phase 1/2	C00-D48	Diffuse large B-cell lymphoma	SPHK2
PF-3526299	Phase 1	J00-J99	Asthma	SYK
TAK-659	Phase 1/2	C00-D48	Acute myeloid leukemia	SYK
Entospletinib	Phase 2	C00-D48	Haematological malignancies	SYK
PRT-062607	Phase 2	C00-D48	Rheumatoid arthritis	SYK
R-343	Phase 2	J00-J99	Allergic asthma	SYK
Fostamatinib	Phase 3	D50-D89	Immune thrombocytopenic purpura	SYK
Fresolimumab	Phase 2	C00-D48	Renal cancer	TGF-beta
TEW-7197	Phase 1	C00-D48	Solid tumours	TGFBR1
Galunisertib	Phase 2/3	C00-D48	Hepatocellular carcinoma	TGFBR1
Altiratinib	Phase 1	C00-D48	Solid tumours	Trk
AZD-6918	Phase 1	C00-D48	Solid tumours	Trk
DS-6051	Phase 1	C00-D48	Solid tumours	Trk
PLX-7486	Phase 1	C00-D48	Solid tumours	Trk
Entrectinib	Phase 2	C00-D48	Solid tumours	Trk
LOXO-101	Phase 2	C00-D48	Solid tumours	Trk
MK-1775	Phase 2	C00-D48	Gynaecological cancer	WEE1

Supplementary Figure S1: The distribution of 3 types of system profiles of phase 1, 2 and 3 clinical trial kinase targets and that of established targets illustrated by the *boxplot*.

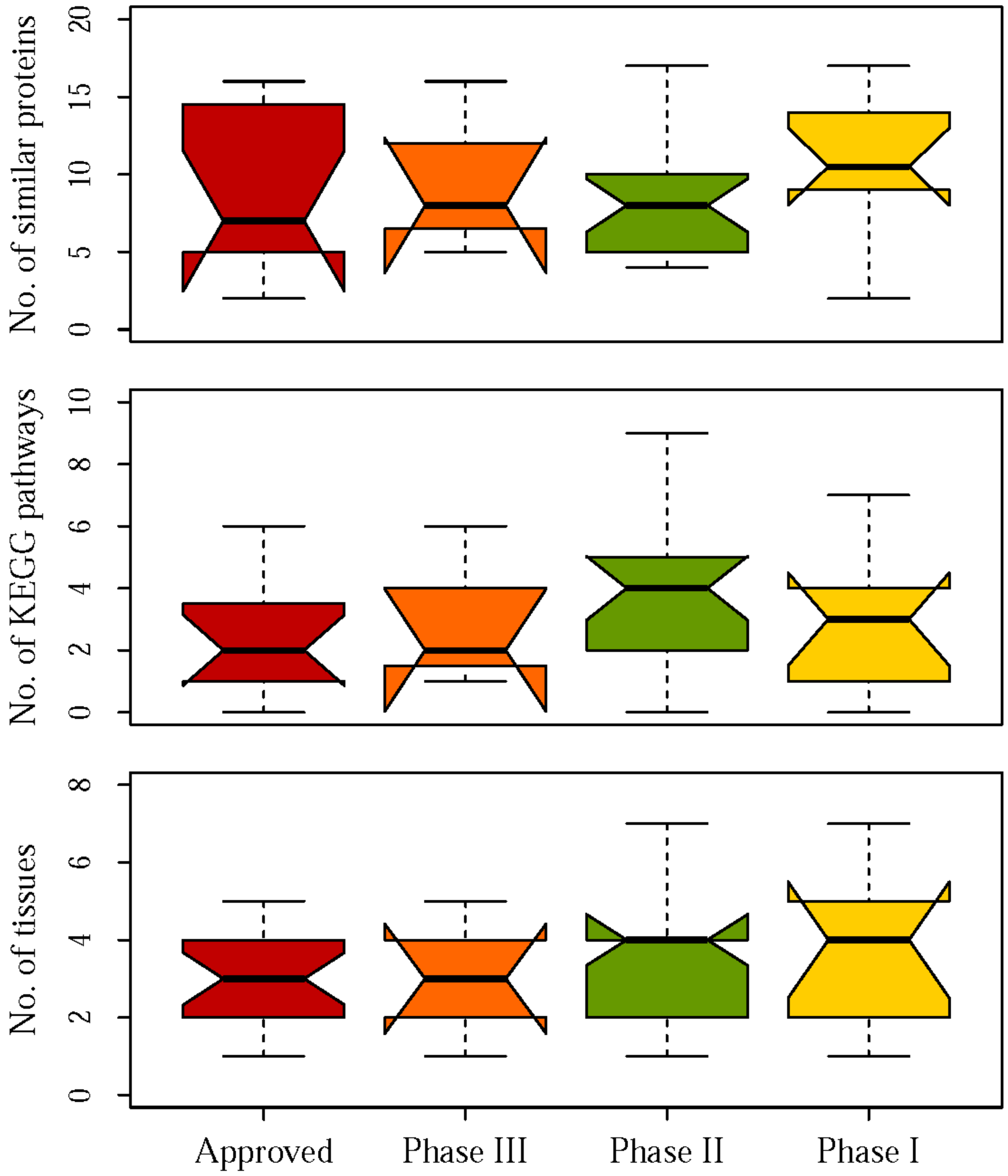

Supplementary Figure S2: A comprehensive drug-target interaction network (part one) including all 46 FDA approved drugs (together with their corresponding 25 established targets) and 239 drugs in clinical trial (including 81 drugs targeting only on 17 established targets, 140 drugs targeting only on 36 clinical trial targets, and 29 multi-target drugs targeting on 13 established and 13 clinical trial targets). Drugs were shown as a round rectangle (approved, phase 3, phase 2 and phase 1 drugs in red, orange, green and yellow, respectively). Established and clinical trial targets were shown by blue and violet ellipses, respectively.

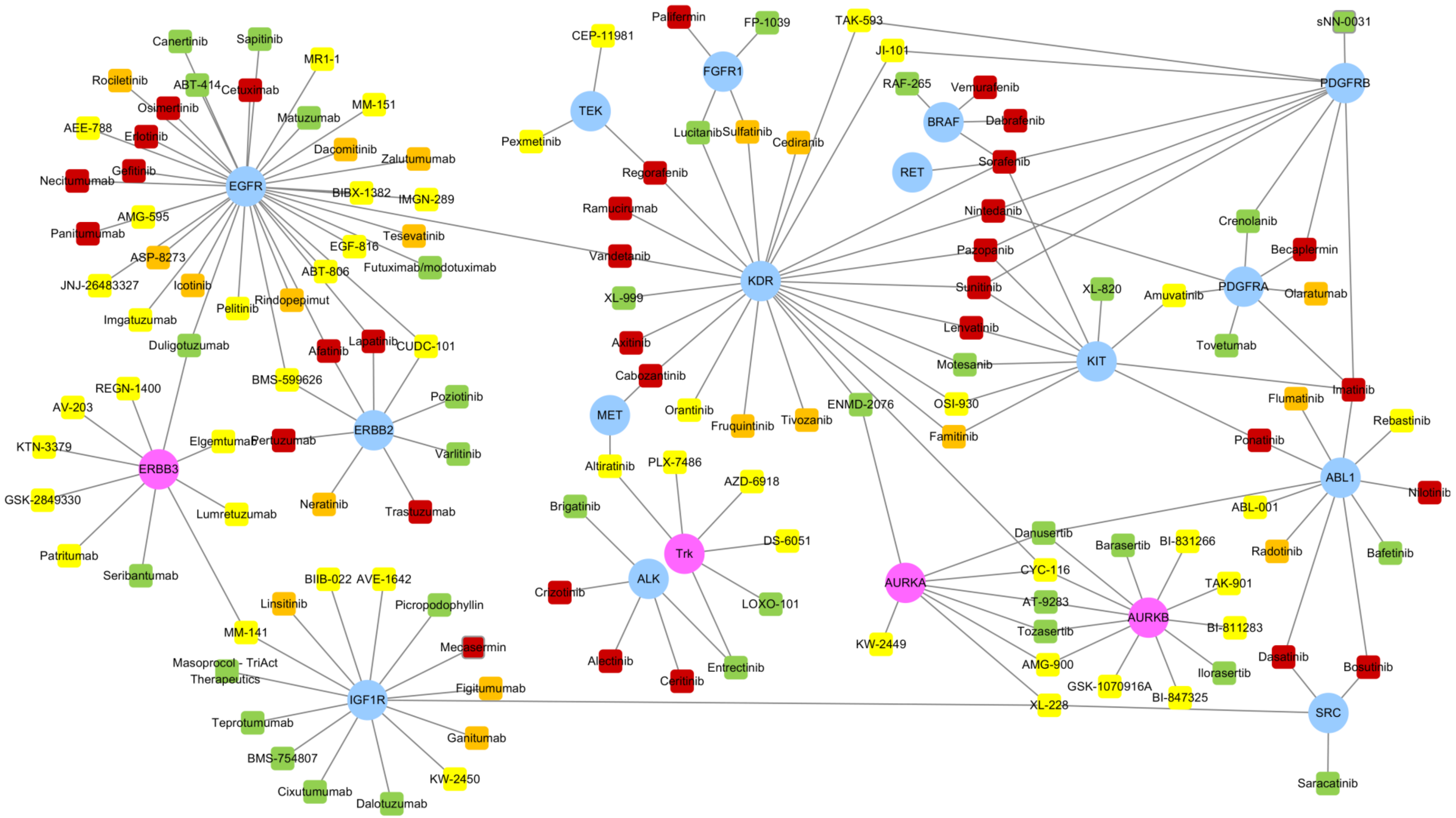

Supplementary Figure S3: A comprehensive drug-target interaction network (part two) including all 46 FDA approved drugs (together with their corresponding 25 established targets) and 239 drugs in clinical trial (including 81 drugs targeting only on 17 established targets, 140 drugs targeting only on 36 clinical trial targets, and 29 multi-target drugs targeting on 13 established and 13 clinical trial targets). Drugs were shown as a round rectangle (approved, phase 3, phase 2 and phase 1 drugs in red, orange, green and yellow, respectively). Established and clinical trial targets were shown by blue and violet ellipses, respectively.

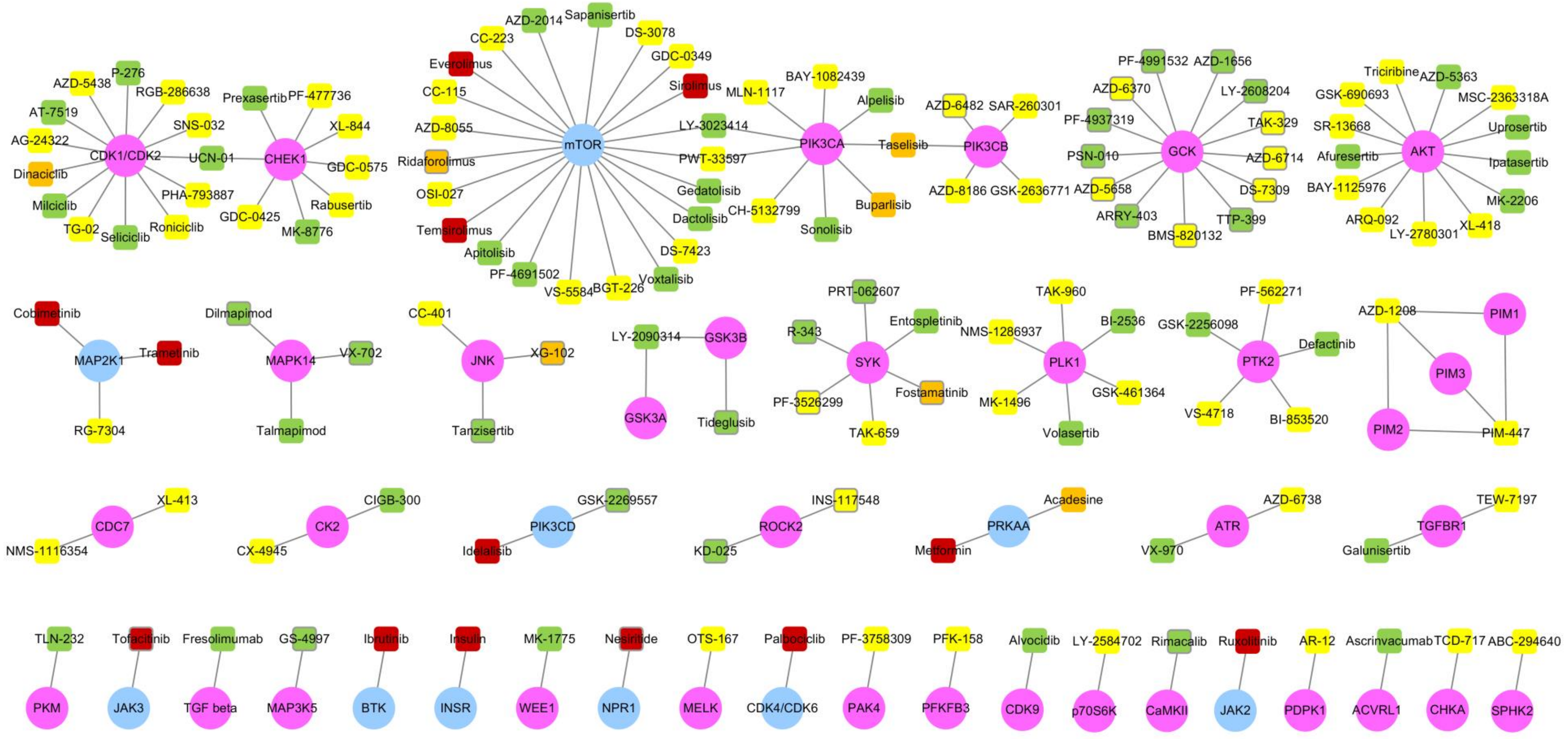

Supplement: Supplementary file 1 — Supplementary material shows the list of drugs targeting on at least one established kinase target together with their detailed therapeutic information (Suppl. Table S1), the list of drugs targeting on at least one clinical trial kinase target together with their detailed therapeutic information (Suppl. Table S2), the distribution of 3 types of system profiles of phase 1, 2 and 3 clinical trial kinase targets and that of established targets (Suppl. Fig. S1) and a comprehensive drug-target interaction network of all 46 FDA approved drugs and 239 drugs in clinical trial (Suppl. Fig. S2, S3). [file 2509385.f1.pdf]
